# Z-Axis Micromachined Tuning Fork Gyroscope with Low Air Damping

**DOI:** 10.3390/mi8020042

**Published:** 2017-02-01

**Authors:** Minh Ngoc Nguyen, Nhat Sinh Ha, Long Quang Nguyen, Hoang Manh Chu, Hung Ngoc Vu

**Affiliations:** 1 International Training Institute for Materials Science, Hanoi University of Science and Technology, Hanoi 100000, Vietnam; ngocminh.utehy@gmail.com (M.N.N.); hasinhnhat.mse@gmail.com (N.S.H.); nqlong@itims.edu.vn (L.Q.N.); 2 Faculty of Electronic and Electrical Engineering, Hung Yen University of Technology and Education, Hung Yen 160000, Vietnam

**Keywords:** Bulk micromachining, Tuning fork gyroscope, Finite element analysis

## Abstract

This paper reports on the design and fabrication of a *z*-axis tuning fork gyroscope which has a freestanding architecture. In order to improve the performance of the tuning fork gyroscope by eliminating the influence of the squeeze-film air damping, the driving and sensing parts of the gyroscope were designed to oscillate in-plane. Furthermore, by removing the substrate underneath the device, the slide-ﬁlm air damping in the gap between the proof masses and the substrate was eliminated. The proposed architecture was analyzed by the finite element method using ANSYS software. The simulated frequencies of the driving and sensing modes were 9.788 and 9.761 kHz, respectively. The gyroscope was fabricated using bulk micromachining technology. The quality factor and sensitivity of the gyroscope operating in atmospheric conditions were measured to be 111.2 and 11.56 mV/°/s, respectively.

## 1. Introduction

Micro-gyroscopes have the advantages of small size, low cost, and suitability for mass production. The micro-gyroscopes can be used in the automotive industry to make anti-rollover systems, antiskid controls, and electronic stability controls [1]. Various applications for consumer equipment range from camera stabilization, cell phone stabilization, virtual reality, and inertial mouse to navigation for portable electronics [2]. The micro-gyroscopes have further applications in robotics and military equipment including inertial navigation for aeronautics and astronautics, and platform stabilization [3]. Mostly, the micro-gyroscopes have vibrating structures suspended above a silicon substrate. Among them, the tuning fork gyroscope (TFG) with an electrostatic drive and capacitive-type sensing is mostly preferred thanks to its reported potential capabilities and advantages such as common-mode rejection and low power consumption [4,5,6,7,8,9]. Generally, for vibratory gyroscopes, the mechanical quality factor (*Q*) of the operating modes is a key performance parameter. In order to achieve higher sensitivity and better rate resolution, the micro-gyroscope needs to be designed with a large sense-mode *Q*. A large drive-mode *Q* is also necessary for improving the bias stability as well as ensuring large drive amplitudes using small drive voltages [10].

The overall mechanical *Q* for an operating mode relates to energy-dissipation mechanisms in the TFG such as air damping (*Q*_Air_), dissipation through the substrate (*Q*_Support_), thermo-elastic damping (*Q*_TED_), and surface loss (*Q*_Surface_) [11], and it is expressed by 1/*Q* = 1/*Q*_Air_ + 1/*Q*_Support_ +1/*Q*_TED_ + 1/*Q*_Surface_. Among them, the effect of air damping is considered as the primary loss mechanism for the device, which operates in atmospheric conditions.

In a typical capacitive detection gyroscope, where a comb-type detection structure is commonly used, the squeeze-film air damping and the slide-film air damping between the proof masses and the substrate usually result in a low *Q*-factor [12,13]. This requires a costly vacuum packaging solution to achieve better sensitivity [14]. Developing high-performance micro-gyroscopes operating at atmospheric pressure is an effective means to reduce the cost.

In recent years, different structure designs have been pursued to improve the performance of micro-gyroscope operating in atmospheric conditions. Geen et al. in Analog Devices developed a single-chip surface micro-machined integrated gyroscope with an atmospheric hermetic package [15]. This device attained a root Allan variance of 50°/h with a full scale range of ±150°/s. Alper et al. reported a silicon-on-insulator microelectromechanical systems (MEMS) gyroscope which operates at atmospheric pressure with a short-term bias stability of 1.5°/s [16]. Che et al. reported on the high *Q*-value for an electrostatic-driven tuning fork micro-machined gyroscope with a bar structure that operates at atmospheric pressure [17]. A single-crystal silicon-based lateral axis tuning fork gyroscope (TFG) with a vertically torsional sensing comb was developed by Guo et al. [12]. However, the quality factor of the sensing mode in this study was rather low due to the squeeze-film air damping between the proof mass and the glass substrate. Hu et al. reported on the sensitivity improvement of a slot-structure micro-gyroscope working in atmospheric conditions through a tunable electrostatic spring constant attained by the triangular-shaped fixed electrodes [18].

In this study, we aim at improving the sensitivity of the angular rate sensor by increasing the quality factor of the driving and sensing modes. We propose a capacitive-type *z*-axis tuning fork gyroscope, which has a freestanding architecture. In order to enhance the sensitivity of the tuning fork gyroscope, the slide-film air damping in the gap between the proof masses and the substrate is eliminated by removing the substrate part underneath the device. The optimal design of the tuning fork gyroscope is carried out by simulating its mechanical behavior with the finite element method (FEM) using ANSYS software. The proposed *z*-axis tuning fork micro-gyroscope has been fabricated and characterized.

## 2. Design and Simulation

**Figure 1:**
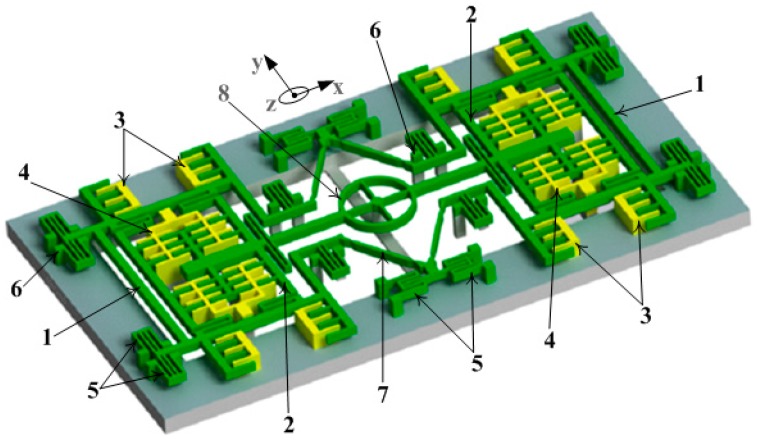
Schematic drawing of the proposed freestanding tuning fork gyroscope: (1) outer frame, (2) inner frame, (3) driving comb electrodes, (4) parallel plate sense electrode, (5) double folded beams, (6) anchors, (7) linear beams and (8) self-rotation ring.

The proposed TFG architecture is shown in Figure 1.

The TFG comprises two proof masses. Each proof mass has an outer frame (1) for driving and an inner one (2) for sensing. The set of driving comb electrodes (3) is attached to the outer frame. This design is such that the masses can oscillate in opposite directions along the *x*-axis under applied electrostatic force in driving mode. The motion of this set obeys the slide-film mechanism, offering highly stable actuation. A pair of parallel plate sense electrode sets (4) is placed symmetrically within each inner frame. Upon the rotation, the Coriolis force excites this frame along an in-plane motion, such that the differential capacitance can be detected. In the design, the double folded beams (5), which have their fixed ends connected to the substrate by anchors (6), are employed for the suspension of the frames. The suspension of the frames is designed to allow the structure to oscillate in two orthogonal modes. In the design, the single folded beams are utilized to join the inner frame with the outer one such that the motion in the orthogonal direction can be easily sensed.

The TFG is operated in the anti-phase drive mode, which excites the masses oscillated in opposite directions. To achieve an anti-phase oscillation in the driving mode, the two masses are coupled with a diamond-shaped coupling spring formed by four linear beams (7). The anti-phase vibration for the sensing mode is guaranteed by using a self-rotation ring (8), which is positioned inside the diamond-shaped coupling spring. This design can be used to stiffen the in-phase resonance of the structure, while leaving the anti-phase compliant. Based on this design the noise is decreased, resulting in the improvement of the sensitivity of the TFG [4].

**Figure 2:**
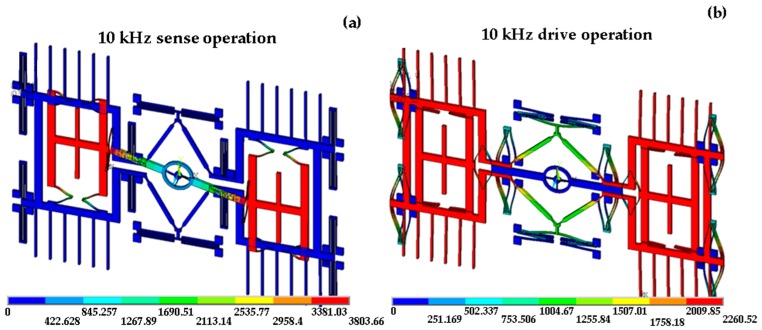
Fundamental operation modes of tuning fork gyroscope obtained by finite element analysis: (**a**) sensing mode and (**b**) driving mode.

The TFG design model was verified by finite element analysis (FEA) using ANSYS software. The dimensional parameters of the proof masses and the suspension beams were investigated to reach the optimally designed mechanical structure of the TFG, since the vibration modes are strongly dependent on these parameters. The modal simulation results for the drive and sense vibration modes of the micro-gyroscope are shown in Figure 2. The resonant frequencies of the driving and sensing modes were determined to be 9.788 and 9.761 kHz, respectively. Thus, the sensor bandwidth was evaluated to be 27 Hz. This bandwidth value satisfies the requirement of both the sensitivity and response time related to the gyroscope operation [19].

Other analysis results of the vibrating modes (not shown in this paper) showed that the higher unwanted modes have frequencies far greater than 15.4 kHz. Thus, there is a difference of about 57.5% between the two sensing and driving modes and the higher parasitic ones. It means that the crosstalk effect can be suppressed. Thus, the designed architecture using the mechanical coupling between driving and sensing proof masses exhibits the desired operation features.

As mentioned above, the structure design of the TFG utilizes a set of interdigitated comb capacitors based on the variable area for large-stroke lateral actuation. For a parallel-plate actuator structure with *N* plates on each side, with thickness *t*, overlap length *L* and plate gap *g*, the total electrostatic drive force in a balanced actuation scheme is given by [19]:

(1) $$F_{\mathrm{drive}}=2N\varepsilon_{0}\frac{tL}{g^{2}}V_{\mathrm{DC}}V_{\mathrm{AC}}$$

where *V*_DC_ is the constant bias voltage applied to the moving fingers and *V*_AC_ is the time-varying voltage applied to the fixed ones. We calculated the electrostatis force with *V*_AC_ = 10 V, *V*_DC_ = 11 V and used them as the input parameters for the simulation.

As shown in the design model, the principle of differential detection is employed by symmetrically placing capacitive electrodes on two opposing sides of the inner mass frames, so that the capacitance change in the electrodes is in opposite directions. It means that a fully differential capacitive bridge is formed. The change in capacitance for an electrode set with *N* fingers on each side can be calculated as:

(2) $$\Delta C=2N\varepsilon_{0}\frac{tL}{g^{2}}Y$$

Where *Y* is the displacement of the sensing electrode in the motion direction.

In fact, for the design of the sensing capacitive structure, there are two gaps, one of which is a smaller one corresponding to *g*, and the other is larger on the opposite side. Here, *g* = 2.5 μm, which is compliant with the resolution of photolithography. In order to optimize the large gap for the arrangement of the sensing fingers, several calculations were performed. Fixing a displacement of 1 µm in the sensing motion, a maximum large gap of 7 µm was determined by relying on the calculation of the change in capacitance *ΔC* using Equation (2). Subsequently, the total gap between the two adjacent fingers on one side of the interdigitated capacitor set is 12.5 µm. The maximum change in capacitance within the above limited condition, calculated by applying Equation (2), is in the range between 0.1 to 0.4 pF.

The structure parameters and calculated characteristics of the TFG are shown in Table 1.

The operation principle of the TFG is based on a tuning fork’s response to the rotation. A *z*-axis input rotation signal perpendicular to the device plane causes a Coriolis-induced transfer of energy to the sense vibration mode. The resulting in-plane displacement along the *y*-axis is sensed capacitively at sense electrodes attached to the inner frame of the proof mass.

The designed micro-gyroscope is aimed at operating in non-vacuum-packaged conditions. In the micro-gyroscope’s performance, air damping is considered to be the dominant damping mechanism which includes slide-film damping and squeeze-film damping. In this study, both the driving and sensing masses were designed to move in-plane, which results in limiting the squeeze-film damping.

To eliminate the limitation of the slide-film air damping in this design, the part of the silicon substrate underneath the movable structures of the TFG was removed, which resulted in a freestanding architecture. This enabled high *Q*-factors for both the driving and sensing modes at atmospheric pressure.

In the mentioned gyroscope performance, air damping is considered to be the dominant damping mechanism which includes slide-film damping and squeeze-film damping. Slide-film damping, or lateral damping, occurs when two plates of an area A, separated by a distance d, slide parallel to each other. The lateral damping coefficient can be expressed as:

(3) $$C_{\mathrm{slide}}=\mu_{\mathrm{eff}}\frac{A}{d}$$

The effective viscosity μ_eff_ is approximated as [20]:

(4) $$\mu_{\mathrm{eff}}=\frac{\mu }{1+2K_{n}+0.2K_{n}^{0.788}e^{-K_{n}/10}}$$

The Knudsen number *K_n_* is the measure of the gas rarefaction effect, which is a function of the gas mean free path λ and the gap *d*:

(5) $$K_{n}=\frac{\lambda }{d}$$

Squeeze-film damping occurs when two parallel plates move toward each other and squeeze the air film in between them. The effective viscosity μ_eff_ in squeeze-film damping is given as [20]:

(6) $$\mu_{\mathrm{eff}}=\frac{\mu }{1+9.638K_{n}^{1.159}}$$

The squeeze-film damping coefficient is calculated as:

(7) $$C_{\mathrm{squeeze}}=\frac{\pi \mu_{\mathrm{eff}}Lw^{3}N}{4h^{3}}+\frac{3\mu_{\mathrm{eff}}LwtpN}{g^{3}}$$

Based on model parameters as shown in Table 1 and the desired working pressure (atmospheric pressure), we calculated the damping coefficients to estimate the quality factors of the TFG.

To obtain a large Coriolis force and a detectable signal in the sense mode, large quality factors in the drive and sense modes are required. In order to get a high quality factor with the micro-gyroscope still operated in air, the damping effect must be reduced. In our micro-gyroscope, both the driving mass and sensing mass are designed to move parallel to the silicon substrate. In the air gaps between the movable part and fixed part of driving and sensing, the dominant viscous damping is slide-film damping between the proof mass and the silicon substrate. By removing a part of the silicon substrate under the movable structure, the slide-film damping was removed, which enabled high *Q*-factors for both the driving and sensing modes at atmospheric pressure. As shown in Figure 1, all the substrate underneath the moving part of the micro-gyroscope was removed.

The quality factor (*Q*) in each operation mode is a critical design parameter of the TFG. A high *Q* in the drive mode is necessary to get a large drive mode vibration amplitude using a small drive voltage, and a high *Q* in the sensing mode contributes to improved rate resolution. Based on the above damping model, we calculated the damping coefficients with the Couette-type model. In the drive mode, the damping coefficient *C*_drive_ is the total of the slide damping caused by the combs’ structure. Meanwhile, in the sensing mode, the damping coefficient *C*_sense_ is related to only the squeeze damping of the sensing combs as variable-gap capacitive detection in the sense mode is applied. Under the atmospheric air condition, *Q*_drive_ and *Q*_sense_ are calculated to be:

(8) $$Q_{\mathrm{drive}}=\frac{\sqrt{k_{\mathrm{drive}}M_{\mathrm{drive}}}}{C_{\mathrm{drive}}}$$

(9) $$Q_{\mathrm{sense}}=\frac{\sqrt{k_{\mathrm{sense}}M_{\mathrm{sense}}}}{C_{\mathrm{sense}}}$$

where k_drive_, *M*_drive_ and *k*_sense_, *M*_sense_ are the stiffness and weight of the driving mode and the sensing mode, respectively. All these parameters were calculated and are shown in Table 1.

Table 2 shows the calculated damping coefficients and quality factors of the drive and sensing modes for the non-freestanding and freestanding TFG.

**Figure 3:**
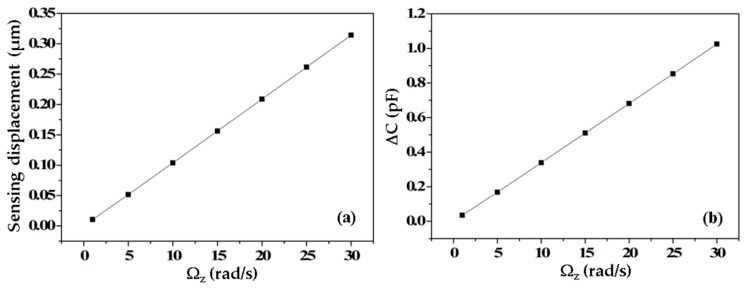
Dependence of the sensing displacement (**a**) and the change in output capacitance Δ*C* (**b**) are investigated as a function of the input angular rate Ω*_z_*.

In order to evaluate the sensitivity of the micro-gyroscope, we simulated the sensing displacement depending on the input angular rate. From this calculated displacement, we derived the sensitivity of the sensor by calculating the change of the capacitance using Equation (2). Here, we input the calculated damping coefficients as shown in Table 2 to investigate the effect of damping on the sensing displacement for the two design cases. When applying an input angular rate of Ω*_z_* = 10 rad/s, the sensing displacement and driving displacement of the TFG are 0.104 and 6.03453 μm, respectively. Compared with the non-freestanding TFG, the displacements in both operated modes at the resonant frequency are higher. The differences in the drive mode and sensing mode are 6% and 48%, respectively. Applying a range of input angular rates on the structure, we received the dependence of the sensing displacement on the input angular rate for the freestanding micro-gyroscope structure, as shown in Figure 3a. Figure 3b shows the dependence of the change in the output capacitance on the input angular rate of the micro-gyroscope. The change in capacitance was calculated using Equation (2). The sensitivity of the two micro-gyroscope structures is shown in Table 2. The results show the sensitivity of the freestanding TFG was evaluated to be 0.034 pF/rad/s, which is 1.6 times higher than that of the non-freestanding TFG design.

## 3. Fabrication

**Figure 4:**
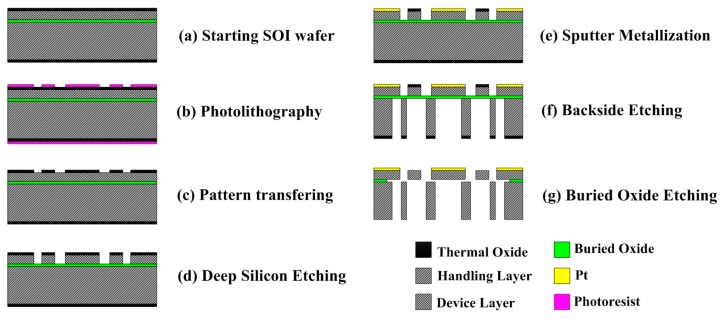
Fabrication process of the proposed tuning fork gyroscope. (**a**) Starting SOI wafer; (**b**) photolithography; (**c**) pattern transferring; (**d**) deep silicon etching; (**e**) sputter metallization; (**f**) backside etching; (**g**) buried oxide etching.

The proposed tuning fork micro-gyroscope was fabricated by bulk micro-machining technology. The main fabrication steps are shown in Figure 4. A 2 cm × 2 cm silicon-on-insulator (SOI) wafer with a 30-µm-thick device layer and a 2 µm buried silicon dioxide layer was used for fabricating the micro-gyroscope. The SOI wafer was first cleaned by a standard cleaning process and thermally oxidized at 1100 °C for 1 h to form a protecting mask for the dry etching process (Figure 4a). Next, the gyroscope patterns were transferred to the SOI wafer surface by photolithography and developing processes (Figure 4b,c). The DRIE process was then performed to a depth of 30 µm to reach the buried dioxide layer of the SOI wafer (Figure 4d). After the etching process, Au electrodes were defined using the lift-off process (Figure 4e). Removing the substrate underneath the device layer was carried out by back-side photolithography and an additional back-side etching step. Finally, a vapor HF etching process was used to etch the SiO_2_ layer underneath the device layer and to release the movable electrodes, beams, and proof masses. The microstructures of the fabricated micro-gyroscope were inspected by scanning electron microscopy (SEM).

**Figure 5:**
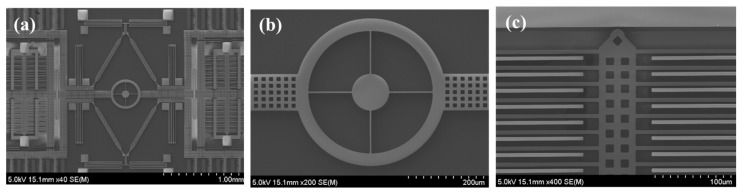
SEM images of a fabricated tuning fork gyroscope: (**a**) TFG chip, (**b**) close-up of the self-rotation ring, and (**c**) sensing comb electrodes.

Figure 5a–c show the whole top view of the TFG, a close-up of the self-rotation ring, and the sensing comb electrodes, respectively. The components of the micro-gyroscope are defined well by the proposed fabrication process.

## 4. Device Characterization and Discussion

**Figure 6:**
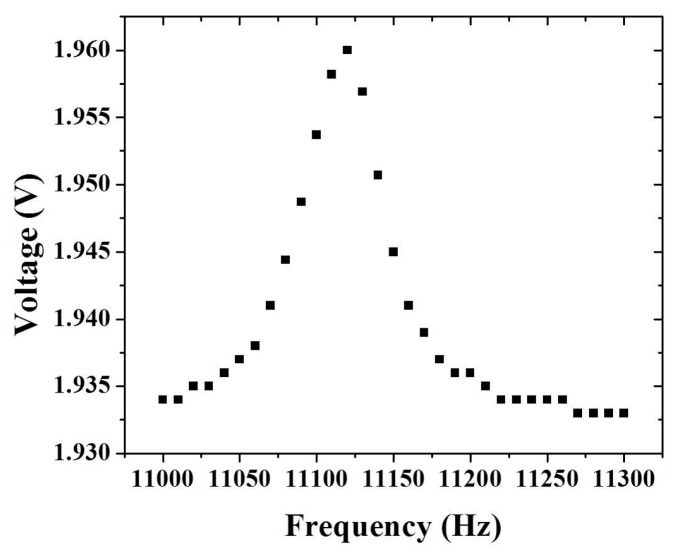
Frequency response of TFG sensing mode.

The frequency and angular rate response of the fabricated tuning fork gyroscope were investigated in atmospheric conditions. Figure 6 shows the measured frequency response of the sensing mode of the TFG. The resonant frequency was about 11.125 kHz. From this, the quality factor of the micro-gyroscope sensing mode was evaluated to be 111.2. The resonant frequency and quality factor of the micro-gyroscope drive mode were 11.250 kHz and 349, respectively. The measured results of the resonant frequencies were different from the simulated results, which is the result of the change of the spring beam width caused by the DRIE process and the holes designed on the micro-gyroscope structure, which caused the mass of sensor’s proof mass to decrease. In our case, the drive and sensing mode frequencies had a mismatch of about 125 Hz, corresponding to the sensor bandwidth. This result satisfies the requirement to optimize the sensitivity and bandwidth [4].

**Figure 7:**
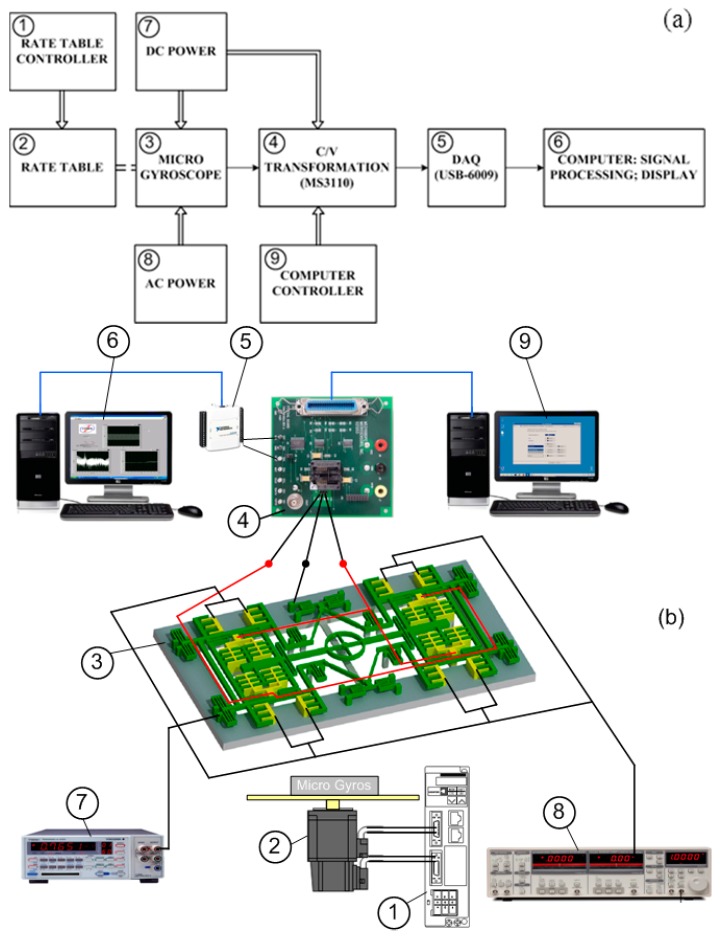
The measurement system is setup for characterizing TFG: (**a**) block diagram and (**b**) experimental setup; the measurement system includes (1) rate table controller; (2) rate table; (3) micro-gyroscope; (4) C/V transformation; (5) DAQ; (6) computer for signal processing and display; (7) DC power; (8) AC power; (9) computer controller.

Figure 7 shows the block diagram and the experimental setup of the measurement system for the TFG angular rate characterization. The atmosphere packaged gyroscope was investigated by coupling the angular rate on a rotation table with a controllable speed by programming.

To excite the micro-gyroscope, an alternating current (AC) voltage of 10 V, with the driving frequency fixed to the resonance frequency of the driving mode at 11.25 kHz, was applied to the movable comb electrode. A direct current (DC) bias of 5 V was applied to the stationary electrode. In order to supply the voltage for the driving comb electrode and detect the capacitance change of the sensing comb structure, the micro-gyroscope was bonded on a printed circuit board (PCB). Wiring from the comb electrodes to the PCB was carried out by using an ultrasonic wedge bonding machine. The capacitance change was transformed to the MS3110 capacitance readout circuit. MS3110 is a commercial product which is used for differential capacitance sensing. This is a preferred C-V readout product due to its stability and detective ability in the fF range. The output of MS3110 is voltage which is proportional to the change in the capacitance of the sensing comb electrodes. The signal was processed by a low-pass filter amplifier and transformed into a personal computer (PC) via the data acquisition (DAQ) module USB6009. This module was used to interface the output signal from the MS 3110 circuit board with the PC. The signal processing was performed by using LabVIEW software.

**Figure 8:**
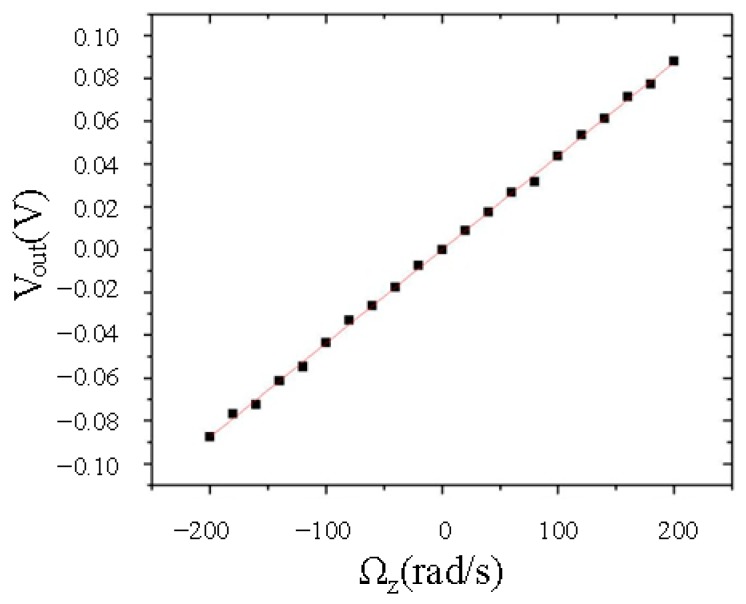
The output voltage *V*_out_ of the TFG is investigated as a function of the input angular rate Ω*_z_*.

Figure 8 shows the measured output signal versus the input angular rate. The output voltage was linearly proportional to the input angular rate in the range from −200°·s^−1^ to 200°·s^−1^. The sensitivity of the gyroscope was determined to be 11.56 mV/°/s.

## 5. Conclusions

A *z*-axis tuning fork micro-gyroscope which has a freestanding and anti-phase controlled architecture was designed and fabricated. The performance of the designed tuning fork gyroscope was improved by limiting the influence of the squeeze-film air damping and slide-film air damping due to the in-plane operation of the driving and sensing modes of the sensor and the freestanding architecture as well. The influence of the crosstalk effect caused by higher parasitic modes on the operating modes was eliminated. The designed micro-gyroscope was fabricated by bulk micro-machining technology. The quality factors of the micro-gyroscope sensing and driving modes operated in atmospheric condition were 111.2 and 349, respectively. The bandwidth was measured to be 125 Hz. The sensitivity of the tuning fork micro-gyroscope was measured to be 11.56 mV/°/s.

Although the tuning fork micro-gyroscope is the most challenging type of transducer in the micro-world, its advantage of high sensitivity makes it have great potential for a variety of applications in everyday life. For our proposed device, one robust structure for improving the sensitivity the micro-gyroscope operated in atmospheric conditions was introduced and verified. However, impact factors such as long-term operation and environment temperature on the operation of the sensor were also investigated. In addition, integration with accelerometers in an inertial measurement unit is also of interest for further development.

## Figures and Tables

**Table 1:** Design parameters of the micro-gyroscope.

Parameters	Dimensions/Performance
Length of driving comb finger (*l*_dcl_)	30 μm
Driving comb overlap length (*L*_dc_)	10 μm
Length of sensing comb finger (*l*_scl_)	100 μm
Sensing comb overlap length (*L*_sc_)	80 μm
Gap between comb fingers (*g*_c_)	2.5 μm
Gap between proof mass and substrate (*h*)	4 μm
Device thickness (*t*)	30 μm
Number of driving comb (*N*_dc_)	1584
Number of sensing comb (*N*_sc_)	480
Sensing area (*A*_sens_)	8.844 × 10^−7^ m^2^
Driving area (*A*_driv_)	1.685 × 10^−6^ m^2^
Driving mass (*M*_driv_)	1.17 × 10^−7^ kg
Sensing mass (*M*_sens_)	6.18 × 10^−8^ kg
Driving mode stiffness (*k*_driv_)	468.471 N/m
Sensing mode stiffness (*k*_sens_)	240.054 N/m
*F*_driv_	134.64 μN

**Table 2:** Damping coefficient, quality factor, and sensitivity of the drive and sensing mode of the non-freestanding and freestanding TFG for comparison.

TFG type	*C*_drive_ (kg/s)	*C*_sense_ (kg/s)	*Q*_drive_	*Q*_sense_	*S* (pF/rad/s)
Non-freestanding TFG	4.5 × 10^−5^	4.59 × 10^−5^	250	84.7	0.021
Freestanding TFG	2.95 × 10^−5^	4.24 × 10^−5^	381	91.8	0.034
